# *Strongyloides stercoralis* infection: an underlying cause of invasive bacterial infections of enteric origin. Results from a prospective cross-sectional study of a northern Italian tertiary hospital

**DOI:** 10.1007/s15010-023-02072-1

**Published:** 2023-07-18

**Authors:** Giulia Gardini, Guenter Froeschl, Francesca Gurrieri, Maria Antonia De Francesco, Chiara Cattaneo, Valentina Marchese, Giorgio Piccinelli, Silvia Corbellini, Chiara Pagani, Marzia Santagiuliana, Benedetta Fumarola, Maurizio Gulletta, Francesca Perandin, Francesco Castelli, Alberto Matteelli

**Affiliations:** 1 Division of Infectious and Tropical Diseases, University of Brescia and ASST Spedali Civili of Brescia, Brescia, Italy; 2 Division of Infectious Diseases and Tropical Medicine, Medical Center of the University of Munich (LMU), Munich, Germany; 3 Department of Microbiology and Virology, University Hospital of Brescia, Brescia, Italy; 4 Division of Hematology, ASST Spedali Civili of Brescia, Brescia, Italy; 5 Postgraduate School of Internal Medicine, University of Brescia, Brescia, Italy; 6 Department of Infectious-Tropical Diseases and Microbiology, IRCCS Sacro Cuore Don Calabria Hospital, Negrar di Valpolicella, Verona, Italy

**Keywords:** *Strongyloides stercoralis*, Strongyloidiasis, Intestinal bacteria, Intestinal parasitosis, Intestinal helminths, Enteric sepsis

## Abstract

**Purpose of the study:**

We assessed the prevalence of *S. stercoralis* in a cohort of inpatients with invasive bacterial infections of enteric origin to investigate whether the parasite may facilitate these bacterial infections even in the absence of larval hyperproliferation.

**Methods:**

We performed a prospective cross-sectional study in a hospital in northern Italy. Subjects admitted due to invasive bacterial infection of enteric origin and potential previous exposure to *S. stercoralis* were systematically enrolled over a period of 10 months. *S. stercoralis* infection was investigated with an in-house PCR on a single stool sample and with at least one serological method (in-house IFAT and/or ELISA Bordier). Univariate, bi-variate and logistic regression analyses were performed.

**Results:**

Strongyloidiasis was diagnosed in 14/57 patients (24.6%; 95% confidence interval 14.1–37.8%) of which 10 were Italians (10/49, 20.4%) and 4 were migrants (4/8, 50.0%). Stool PCR was performed in 43/57 patients (75.4%) and no positive results were obtained.

Strongyloidiasis was found to be significantly associated (*p* ≤ 0.05) with male gender, long international travels to areas at higher endemicity, deep extra-intestinal infectious localization and solid tumors. In the logistic regression model, increased risk remained for the variables deep extra-intestinal infectious localization and oncologic malignancy.

**Conclusions:**

Our findings suggest a new role of chronic strongyloidiasis in favoring invasive bacterial infections of enteric origin even in the absence of evident larval dissemination outside the intestinal lumen. Further well-designed studies should be conducted to confirm our results, and possibly establish the underlying mechanisms.

**Supplementary Information:**

The online version contains supplementary material available at 10.1007/s15010-023-02072-1.

## Background

*Strongyloides stercoralis* is a nematode (roundworm) transmitted to humans with the passage of filariform larvae (L3, up to 600 µm long) from contaminated soil through intact skin of a host.

Its life cycle is very peculiar, as a free-living sexual cycle and an asexual cycle inside the host are present [1]. The parasite cycle inside the host with the autoinfection phenomenon permits adult females (2–3 mm long) to persist potentially life-long in the hosts’ small intestinal submucosa, where they deposit eggs. From eggs, larvae hatch (L1, 180–380 µm long) that can be excreted in stool and/or enter the autoinfection cycle after conversion to L3 that penetrate the host’s large intestinal mucosa or perianal skin.

In high-income countries as Italy, migrants and international travelers from areas at higher endemicity, as well as elderly autochthonous individuals represent the three main categories at risk for the infection [2–4]. Autochthonous infections in young Italians without a relevant travel history are rare [5].

Complicated *S. stercoralis* infection involves mainly the immunocompromised population and is defined by either hyperinfection or dissemination, where a great amount of larvae invades host organ systems. The mortality of these forms can reach, respectively, 60% and 100% without treatment [6]. It is widely reported in literature how complicated strongyloidiasis may determine concomitant bacterial extra-intestinal infections of enteric origin. Enteric bacteria are not only transported directly on the surface of *S. stercoralis* L3 crossing larvae, but can also escape the gut lumen through parasite-determined intestinal ulcers [7, 8].

To test the hypothesis that chronic strongyloidiasis may represent a facilitating factor for bacterial extra-intestinal infections of enteric origin even in the absence of larval hyperproliferation, we conducted a prospective cross-sectional study to measure the prevalence of strongyloidiasis in a cohort of patients with severe bacterial extra-intestinal infection whose focus of origin was the gastrointestinal tract (e.g. sepsis, deep abscesses, meningitis, endocarditis, spondylodiscitis, pneumonia) and with epidemiological risk factors for this helminthiasis. The secondary purpose of the study was to identify additional epidemiological and clinical risk factors for strongyloidiasis.

## Methods

### Design and setting of the study

We conducted a prospective cross-sectional monocentric study at the ASST Spedali Civili of Brescia University Hospital, affiliated to the University of Brescia (Lombardy Region, North of Italy) from January to October 2021.

### Study population and sample size

Eligible patients were inpatients admitted to the Infectious and Tropical Diseases, Hematology or Internal Medicine divisions due to bacterial extra-intestinal infection whose focus of origin was the gastrointestinal tract, and at least one of the following epidemiological risk criteria for strongyloidiasis: (1) Italians born in 1952 or earlier; (2) international travelers with a life-time stay of more than 14 cumulative days in countries with an estimated prevalence of strongyloidiasis ≥ 5% (estimated country-specific prevalence is reported in the supplementary material S1 of the manuscript of Buonfrate et al. [9]); (3) migrants coming from countries with an estimated prevalence of strongyloidiasis ≥ 5% [9]).

Patients were excluded if one of the following criteria was present: (1) age < 18 years; (2) denied or unable to sign the informed consent form; (3) focus of infection different from gastrointestinal tract (e.g. urinary apparatus, central venous catheter, cutaneous lesions/ulcers).

Since the background prevalence of *S. stercoralis* in the study population was not known, we decided to consecutively enroll patients over a pre-defined period of time.

### Study procedures

Enrolled patients provided: (1) a written interviewer-administered questionnaire (see supplementary material 1) on socio-demographic characteristics, exposure and clinical factors. Medical records were consulted to retrieve clinical information; (2) a single stool sample for in-house PCR for *S. stercoralis* (sensitivity 86.4% (19/22) when larvae are detected with microscopy after concentration by Baermann method; sensitivity 43.8% (14/32) when larvae are not detectable with microscopy after concentration by Baermann method but with coproculture; specificity 100% [10]); (3) a blood sample to perform at least one serological test for *S. stercoralis* (in-house IFAT performed by laboratory of tropical microbiology of Sacro Cuore Don Calabria Negrar Hospital (Verona, Italy) (sensitivity 93.9%; specificity 92.2%) and/or ELISA Bordier kit (sensitivity 89.5%; specificity 98.3%;) [11]).

### Data management

Data were entered anonymously into a Microsoft Excel spreadsheet (Microsoft Corp, USA).

### Analysis and statistical methods

Data analysis was performed using STATA software, Version SE14 (STATA Corp, USA). Variables are presented as proportions, stratified by *Strongyloides* status. The association between socio-demographic and clinical and behavioral parameters with *Strongyloides* infection was calculated using Chi2 test. A threshold of significance was defined as *α* = 0.05, where the null hypothesis is to be rejected when *p* ≤ 0.05. In tabulations with more than 20% small cell sizes (frequency < 5), Fisher’s exact test was applied. For comparison of age distribution between independent groups as non-normally distributed continuous data, the Wilcoxon rank sum test was applied. The 95% confidence interval (CI) of the overall proportion of *Strongyloides*-positive cases in the entire dataset was calculated based on the assumption of a binomial distribution. In the analytical part, odds ratios were calculated using a logistic regression model with backward selection of variables by applying the Wald test with a *p* value cut-off point at 0.5.

## Results

A total of 57 patients were eligible and all accepted to participate in the study.

Of them, 45 (79.0%) were admitted to the Infectious Diseases division, 8 (14.0%) to the Hematology division and 4 (7.0%) to the Internal Medicine division.

The most frequent single eligibility criterion for inclusion was being an Italian citizen born in 1952 or earlier (34/57; 59.6%), followed by being a migrant (8/57; 14.0%) or having traveled to areas with estimated prevalence for strongyloidiasis ≥ 5% for at least 14 cumulative days (3/57; 5.3%). Twelve patients (21.1%) had multiple inclusion criteria (elderly Italians with relevant travel history).

Of the enrolled patients, 49/57 (86.0%) were Italian citizens; 3/57 (5.3%) were from other European countries (Albania, Russia, former Yugoslavia); 1/57 (1.8%) came from China and 4/57 (7.0%) from Africa (Morocco, Senegal, Ghana). Almost all Italian citizens were born in northern Italy, except one from central Italy and one born in Belgium. All enrolled patients resided in northern Italy at the time of study enrollment. Overall, 40/57 (70.2%) were males; the median age was 76 years (IQR 72–82).

**Fig. 1:**
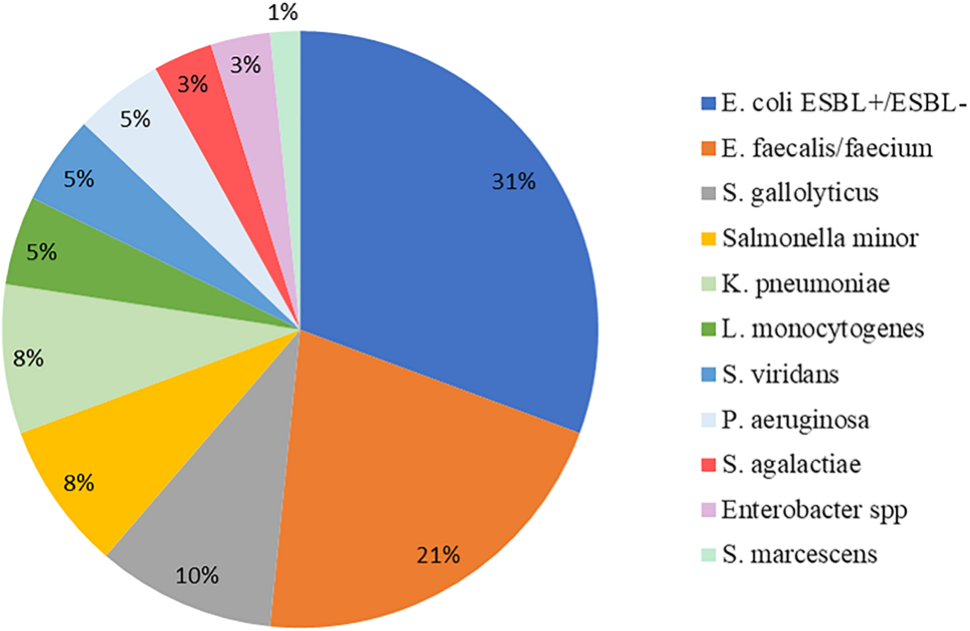
Bacteria isolated from blood or extra-intestinal site of infection

In total, 44/57 (77.2%) enrolled patients presented an invasive infection by Enterobacteriaceae, whereas 14/57 (24.6%) by other enteric bacteria; most frequently isolated bacteria were *E. coli*, *E. faecalis/faecium* and *S. gallolyticus* (for details, see Fig. 1).

Most had a bloodstream infection (54/57, 94.7%), while 14/57 (24.6%) presented deep abscesses and/or other deep bacterial localizations (spondylodiscitis, endocarditis, aortitis, pneumonia), 2/57 (3.5%) had culture-positive meningitis. Only 3/57 (5.3%) had an eosinophil count ≥ 500 cells/µl at the time of admission (2 Italians and one from Morocco). One patient (1.8%) died because of hemorrhagic shock due to intestinal bleeding.

Chronic or recurrent suggestive symptoms for strongyloidiasis were referred in few cases: pruritus in 7/55 (12.7%), gastrointestinal discomfort in 3/55 (5.5%), respiratory symptoms in 3/55 (5.5%) and skin lesions in 1/55 (1.8%).

No one had clinical characteristics suggestive for disseminated strongyloidiasis.

Strongyloidiasis was diagnosed in 14/57 (24.6%; 95% CI 14.1–37.8%) patients (positivity of at least one diagnostic technique), of which 10 were Italians (10/49, 20.4%) and 4 were migrants (4/8, 50.0%) (from China, Morocco, Albania and ex-Yugoslavia). Stool PCR was negative for all tested patients (43/57, 75.4%). IFAT and ELISA were both positive in 2/55 (3.6%) individuals. IFAT alone was positive in 12/55 (21.8%) subjects, while ELISA in 4/57 (7.0%). Further details are present in Table 1.

**Table 1:** *S. stercoralis* testing result constellations

Method	Proportion performed	Positive result	Negative result
At least one positive result in any method	57/57 (100.0%)	14/57 (24.6%)	43/57 (75.4%)
Stool PCR	43/57 (75.4%)	0/43 (0.0%)	43/43 (100.0%)
IFAT	55/57 (96.5%)	12/55 (21.8%)	43/55 (78.2%)
ELISA	57/57 (100.0%)	4/57 (7.0%)	53/57 (93.0%)
IFAT and ELISA	55/57 (96.5%)	2/55 (3.6%)	53/55 (96.4%)

Strongyloidiasis was found to be significantly associated with male gender (*p* = 0.04), international travels with duration ≥ 14 continuative days to areas with estimated prevalence ≥ 5% for the parasitosis (*p* = 0.04), deep extra-intestinal infectious localization (*p* = 0.01), solid tumors (*p* = 0.01) and the category “other gastrointestinal disorder” (*p* = 0.01), that included alcoholic liver cirrhosis, gastric bypass, intestinal occlusion and *pseudomelanosis coli*. Presence of a hematologic malignancy was inversely correlated with *S. stercoralis* (*p* = 0.01). For details, see Tables 2, 3 and 4.

**Table 2:** Socio-demographic characteristics and exposure of study population

Characteristic	Strongyloides positive	Strongyloides negative	Total	Test *p* value
Total	14/57 (24.6%)	43/57 (75.4%)		
Sex male	13/40 (32.5%)	27/40 (67.5%)	40/57 (70.2%)	0.04*
Age (median; IQR; in years)	79 (65–84)	76 (73–82)	76 (72–82)	0.82**
Heterosexuality	14/54 (25.9%)	40/54 (74.1%)	54/54 (100.0%)	NA
Born outside Italy^#^	4/9 (44.4%)	5/9 (55.6%)	9/57 (15.8%)	0.20*
Primary education (age 6–11)	6/26 (23.1%)	20/26 (76.9%)	26/55 (47.3%)	0.86
Lower secondary education (age 11–14)	5/20 (25.0%)	15/20 (75.0%)	20/55 (36.4%)	
Upper secondary education (age 14–19)	2/7 (28.6%)	5/7 (71.4%)	7/55 (12.7%)	
Higher education	1/2 (50.0%)	1/2 (50.0%)	2/55 (3.6%)	
Working in primary sector (extraction of raw material)	4/21 (19.1%)	17/21 (81.0%)	21/56 (37.5%)	0.53*
History of frequent barefoot walking	10/34 (29.4%)	24/34 (70.6%)	34/55 (61.8%)	0.39
Past intestinal worms infection	6/24 (25.0%)	18/24 (75.0%)	24/55 (43.6%)	0.95
Past close contact with animals	13/50 (26.0%)	37/50 (74.0%)	50/55 (90.9%)	0.77
Present close contact with animals	5/30 (16.7%)	25/30 (83.3%)	30/55 (54.6%)	0.10
Past rural activity	11/39 (28.2%)	28/39 (71.8%)	39/55 (70.9%)	0.47
Present rural activity	8/33 (24.2%)	25/33 (75.8%)	33/55 (60.0%)	0.80
International travels (outside Italy)	12/46 (26.1%)	34/46 (73.9%)	46/56 (82.1%)	0.69
International travels to areas with estimated prevalence for strongyloidiasis > = 5%	10/29 (34.5%)	19/29 (65.5%)	29/46 (63.0%)	0.16*
International travels to areas with estimated prevalence for strongyloidiasis > = 5% if > = 14 continuative days	9/21 (42.9%)	12/21 (57.1%)	21/28 (75.0%)	**0.04**
International travels to areas with estimated prevalence for strongyloidiasis > = 5% if < 14 continuative days	1/8 (12.5%)	7/8 (87.5%)	8/18 (44.4%)	1.00*

All measures of association were tested by applying the chi2 test, except in * where Fisher’s exact test was applied and ** where the Wilcoxon rank sum test was applied. NA: not applicable test of association due to 0 observations in a category. *p* values ≤ 0.05 are indicated in bold

*IQR* interquartile range

^#^Included here 1 individual born in Belgium and immediately after birth transferred to Italy, also bearing an Italian citizenship. This person is considered further on part of the sub-group of Italians

**Table 3:** Clinical variable of study population

Characteristic	Strongyloides positive	Strongyloides negative	Total	Test *p* value
Total	14/57 (24.6%)	43/57 (75.4%)		
Enterobacteriaceae	10/44 (22.7%)	34/44 (77.3%)	44/57 (77.2%)	0.55
Non-Enterobacteriaceae	4/14 (28.6%)	10/14 (71.4%)	14/57 (24.6%)	0.73*
Bloodstream infection	12/54 (22.2%)	42/54 (77.8%)	54/57 (94.7%)	0.08
Meningitis	0/2 (0.0%)	2/2 (100.0%)	2/57 (3.5%)	1.00*
Deep abscesses	3/5 (60.0%)	2/5 (40.0%)	5/57 (8.8%)	0.09*
Other extra-intestinal infection site (spondylodiscitis, endocarditis, aortitis, pneumonia)	5/11 (45.5%)	6/11 (54.6%)	11/57 (19.3%)	0.07
Deep abscesses and/or other extra-intestinal infection site	7/14 (50.0%)	7/14 (50.0%)	14/57 (24.6%)	**0.01**
Eosinophil count > = 500 cells/µl	0/3 (0.0%)	3/3 (100.0%)	3/57 (5.3%)	0.57*
Itching	2/7 (28.6%)	5/7 (71.4%)	7/55 (12.7%)	1.00*
Skin lesions	0/1 (0.0%)	1/1 (100.0%)	1/55 (1.8%)	1.00*
Gastrointestinal discomfort	1/3 (33.3%)	2/3 (66.7%)	3/55 (5.5%)	1.00*
Respiratory symptoms	0/3 (0.0%)	3/3 (100.0%)	3/55 (5.5%)	0.56*
Outcome death	1/1 (100.0%)	0/1 (0.0%)	1/57 (1.8%)	0.43*

All measures of association were tested by applying the chi2 test, except in * where Fisher’s exact test was applied. *p* values ≤ 0.05 are indicated in bold

**Table 4:** Investigated co-morbidities of study population

Characteristic	Strongyloides positive	Strongyloides negative	Total	Test *p* value
Total	14/57 (24.6%)	43/57 (75.4%)		
Hematologic malignancy	1/22 **(4.6%)**	21/22 (95.5%)	22/57 (38.6%)	0.01*
Oncologic malignancy	6/11 (54.6%)	5/11 (45.5%)	11/57 (19.3%)	**0.01**
Rheumatological disorder	1/4 (25.0%)	3/4 (75.0%)	4/57 (7.0%)	1.00*
Transplant recipient	0/1 (0.0%)	1/1 (100.0%)	1/57 (1.8%)	1.00*
Known HIV or HTLV-1 infection	0	0	0/57	NA
Diabetes	4/12 (33.3%)	8/12 (66.7%)	12/57 (21.1%)	0.46*
Dialysis	2/4 (50.0%)	2/4 (50.0%)	4/57 (7.0%)	0.25*
Terminal renal insufficiency	0/2 (0.0%)	2/2 (100.0%)	2/57 (3.5%)	1.00*
GI disorders	8/30 (26.7%)	22/30 (73.3%)	30/57 (52.6%)	0.70
Other GI disorder^#^	4/5 (80.0%)	1/5 (20.0%)	5/57 (8.8%)	0.01*
Mental disorder	2/14 (14.3%)	12/14 (85.7%)	14/47 (24.6%)	0.48*
Alcohol abuse	4/11 (36.4%)	7/11 (63.6%)	11/57 (19.3%)	0.44*
Systemic steroids	4/20 (20.0%)	16/20 (80.0%)	20/57 (35.1%)	0.75*
Chemotherapy	2/16 (12.5%)	14/16 (87.5%)	16/57 (28.1%)	0.31*
Present COVID-19	1/4 (25.0%)	3/4 (75.0%)	4/57 (7.0%)	1.00*

*GI* gastrointestinal

^#^Included disorders: alcoholic liver cirrhosis, gastric bypass, intestinal occlusion and *pseudomelanosis coli*

All measures of association were tested by applying the chi2 test, except in * where Fisher’s exact test was applied. NA: not applicable test of association due to 0 observations in a category. *p* values ≤ 0.05 are indicated in bold

In the logistic regression model, increased risk remained with odds ratios > 1 and *p* ≤ 0.05 for the variables deep extra-intestinal infectious localization, oncologic malignancy and other gastrointestinal disorders (see Table 5).

**Table 5:** Results of the logistic regression model

Outcome variable: Strongyloides status	Odds ratio	Standard error	*p* >|*z*|	95% CI lower	95% CI upper
Migrant	5.8	8.6	0.24	0.31	1.1 × 10^2^
Travel to endemic country ≥ 14 days	15	25	0.12	0.51	4.2 × 10^2^
Sepsis	5.4 × 10^–3^	0.015	0.05	2.9 × 10^–5^	1.0
Deep extra-intestinal infectious localization	31	46	0.02	1.6	5.8 × 10^2^
Oncologic malignancy	43	69	0.02	1.8	1.0 × 10^3^
Other GI disorder	3.2 × 10^2^	7.5 × 10^2^	0.02	3.1	3.2 × 10^4^

*CI* confidence interval

## Discussion

To the best of our knowledge, this is the first clinical prospective cross-sectional study that investigates the association between *S. stercoralis* and bacterial extra-intestinal infection of enteric origin in absence of larval hyperproliferation. In literature, we only found a retrospective medical records review of patients with strongyloidiasis performed in Kentucky (USA) [12] and two case reports [13, 14] where authors speculate about this new role of strongyloidiasis.

The seroprevalence of strongyloidiasis in our cohort was higher compared with previous studies on non-hospitalized and non-septic individuals [2], both considering the criterion of one positive method (24.6%) and 2 concordant serological tests (3.6%). This was observed both in Italian and migrant subjects.

Remarkably, our cohort only included three individuals with eosinophilia and all three were negative for strongyloidiasis.

Although eosinophilia has usually been considered a key predictive sign for strongyloidiasis, our data suggest that bacterial invasive infections of enteric origin might also be considered a predictive condition. In our Italian cohort, bacterial invasive infection of enteric origin predicted a strongyloidiasis prevalence of 20.4% (one positive serological result), not different from the 28% prediction of an eosinophil count > 500 cell/µl in a study of elderly Italians tested with IFAT serology [15].

None of our participants showed a positive result by PCR on stool. This may be explained in part by the known intermittency and low parasite load in stool excreted during chronic strongyloidiasis. As a consequence, even molecular amplification techniques suffer from low sensitivity in these cases [16, 17], especially when a single stool sample per patient is analyzed. Instead, this stool result together with clinical patterns corroborate the non-hyperproliferative larval status of our patients.

In our study, we cannot determine the impact of serological cross reactions leading to potentially false-positive results. However, the serological methods we used have high sensitivity and the existing Italian seroprevalence studies share this limitation.

In the bi-variate analysis, the significant association (*p* ≤ 0.05) between strongyloidiasis and the single factor male gender [18] and oncologic neoplasms had already been previously reported [19].

The significant association with international travels for more than 14 continuative days to areas with an estimated strongyloidiasis prevalence ≥ 5% is of interests in terms of travel duration. Current suggestion is to screen for *S. stercoralis* the immunocompetent international traveler who stayed for more than 1 cumulative year in areas of higher endemicity [4]. Our result indicates that shorter staying duration in at risk areas should be considered for screening.

The significant association with deep extra-intestinal localizations of infection could involve two mechanisms, which are not mutually exclusive: the presence of deep abscesses/localizations instead of just a bloodstream infection may correspond to the peculiarity of *Strongyloides* larvae to migrate across organs and also deep connective tissues; in addition, as hypothesized for other helminths [20, 21], migrating larvae may induce granulomas in human organs which in turn may represent a site of bacterial attachment and replication.

We are currently unable to explain fully the statistically significant inverse correlation with hematologic malignancy. None of our patients were screened or treated before for this parasitosis. We can speculate that this population had suffer from lower sensitivity of serological diagnostic tools due to immunosuppression.

The association with the category “other gastrointestinal disorder” should be considered a spurious result considering both the exiguity of patients and heterogeneity of disorders combined under this group.

Eosinophilia and symptoms suggestive of strongyloidiasis were not predictive for this parasitosis in our cohort. Therefore, all our diagnoses, except of that made in one Moroccan immunocompromised man, would have been lost according to current recommended criteria for screening. Of note, acute bacterial infection and septic status can mask a pre-existing eosinophilia in favoring of neutrophilia, as well described in literature [22, 23].

The final logistic regression model confirmed as significant risk factors for the outcome variable *Strongyloides* status the independent variables deep extra-intestinal infectious localization, oncologic malignancy and the compound variable “other GI disorders”. However, the model is surely lacking power due to the limited sample size.

**Fig. 2:**
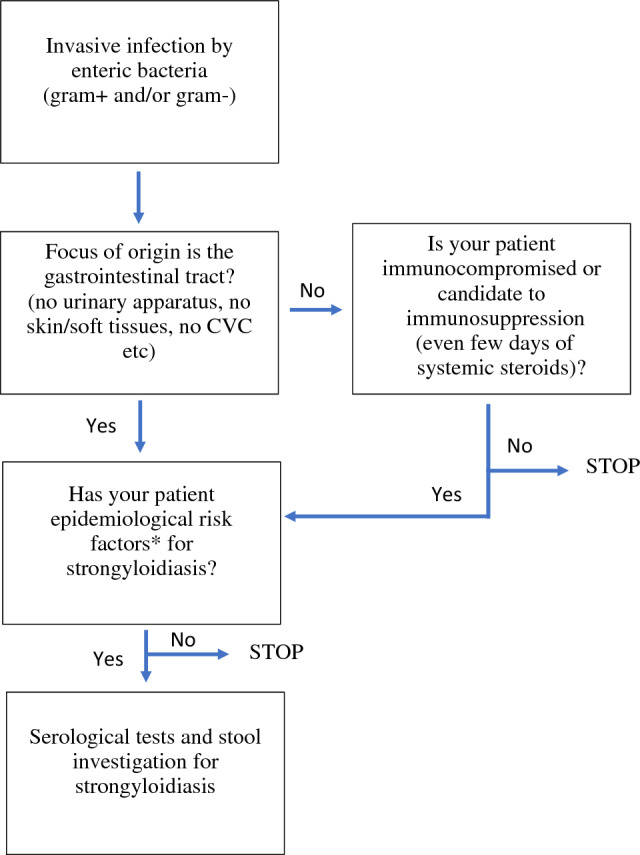
Suggested decisional flow chart for *S. stercoralis* screening in patients with bacterial invasive infection of enteric origin. *Elderly autochthonous Italian (even in the absence of relevant travel history), migrant or traveler with a history of staying ≥ 14 continuative days in areas with an estimated prevalence ≥ 5% for *S. stercoralis*

Based on our results, we recommend to clinicians to consider bacterial extra-intestinal infections of enteric origin, especially in case of deep localizations, as a warning sign for an underlying intestinal strongyloidiasis in patients with epidemiological risk factors for soil-transmitted helminths (see Fig. 2 for suggested decisional flow chart). In these categories, we consider elderly Italians even in the absence of relevant travel history, migrants and international travelers especially when a history of staying ≥ 14 continuative days in areas with an estimated prevalence ≥ 5% for *S. stercoralis* is present.

We speculate that the mechanism for the favoring role of strongyloidiasis on deep bacterial infections could be a transient crossing of low numbers of larvae through the intestinal barrier with concomitant translocation of enteric bacteria. Otherwise, the long persistence of *S. stercoralis* (adult female, eggs, larvae) in the human gut lumen could alter the composition of the bowel microbiome and the local and/or systemic immune response conferring a higher risk to develop enteric sepsis.

Our study has limitations, primarily the small study sample. In addition, our patients represent a population consulting a third-level medical facility; hence, external validity of our findings, e.g. towards primary care settings, may be limited as well.

## Conclusions

The results of our study suggest a new role of chronic strongyloidiasis in favoring extra-intestinal bacterial infections of enteric origin even in the absence of evident larval hyperproliferation and dissemination outside the intestinal lumen. Further well-designed studies should be conducted to confirm our results, eventually establish the underlying mechanisms and the individual and social burden of this phenomenon. In the meantime, both considering the relatively low cost of diagnosis and the harmful potential of undetected strongyloidiasis, we recommend to test for this parasitosis all individuals presenting with invasive bacterial infections of enteric origin if epidemiological risk factors for the parasitosis are present.

### Supplementary Information

Below is the link to the electronic supplementary material.

Supplementary material 1: Written interviewer-administered questionnaire on socio-demographic characteristics, exposure and clinical factors (English version), pdf format. (DOCX 19 KB)

## Data Availability

The dataset used and analyzed during the current study is available from the corresponding author on reasonable request.

## Abbreviations

IQR: Interquartile range
IFAT: Immunofluorescence antibody test
ELISA: Enzyme-linked immunosorbent assay
CI: Confidence interval
PCR: Polymerase chain reaction
OR: Odd ratio
