# Supplementary material for: Strongyloides stercoralis infection: an underlying cause of invasive bacterial infections of enteric origin. Results from a prospective cross-sectional study of a northern Italian tertiary hospital
Source: Infection. 2023 Jul 18;51(5):1541–8. doi: 10.1007/s15010-023-02072-1 (PMC10545639; doi:10.1007/s15010-023-02072-1)
Supplement: Supplementary file 1 — Supplementary material 1: Written interviewer-administered questionnaire on socio-demographic characteristics, exposure and clinical factors (English version), pdf format. (DOCX 19 KB) [file 15010_2023_2072_MOESM1_ESM.docx]

**Supplementary material 1. Written interviewer-administered questionnaire on socio-demographic characteristics, exposure and clinical factors.**

| Name, surname, contact information |  | |
| --- | --- | --- |
| Date of birth |  | |
| Higher level of education |  | |
| Work/Profession |  | |
| City, province, state of birth |  | |
| City, province, state of residence |  | |
| Medium-long staying in other cities, provinces, states of Italy? If yes, where and how long. |  | |
| Number of cohabitants in childhood/adolescence | ≤ 5 □ 6-10 □ > 10 □ | |
| Isolated bacteria, site of infection*  *Report only the infections caused by bacteria whose focus of origin is the gastrointestinal tact | 1. | |
|  | 2. | |
| Eosinophilia (>= 500/µl) | Yes, maximum value: /µl | No □ |
| History of frequent barefoot walking | Yes □ | No □ |
| Travels outside Italy | If yes, indicate where and for each travel specify stay duration (< or >= 14 continuatives days) | Years |
|  |  |  |
| Close contact with animals | If yes, indicate the animal/s | When |
|  |  |  |
| Relevant rural activity | If yes, specify the type (gardening, farming, camping, hunting) | When |
|  |  |  |
| Comorbidities | Immunosuppressive conditions: Yes □ No □  List: | |
|  | Diabetes: Yes □ No □ | |
|  | Gastrointestinal disorders: Yes □ No □  List: | |
|  | Mental disorders: Yes □ No □  List: | |
|  | Alcoholic abuse: Yes □ No □ | |
|  | HTLV-1 infection: Yes □ No □  Ever tested for it? Yes □ No □ | |
| Immunosuppressive drugs | List: | |
| Sexual orientation | Omosexual □ eterosexual □ bisexual □ | |
| History of intestinal worms | If yes, specify when (childhood, adolescence, adulthood): | |
| History of typhus | If yes, specify when (childhood, adolescence, adulthood): | |
| SARS-CoV-2 infection | Present □ Past □ | |
| Suggestive signs/symptoms for strongyloidiasis | 1. Gastrointestinal discomfort: Yes □ No □ | |
|  | 2. Skin lesions: Yes □ No □ | |
|  | 3. Respiratory symtomps: Yes □ No □ | |
|  | 4. Pruritus: Yes □ No □ | |
